# Local Normal Mode Analysis for Fast Loop Conformational Sampling

**DOI:** 10.1021/acs.jcim.2c00870

**Published:** 2022-09-13

**Authors:** José Ramón López-Blanco, Yves Dehouck, Ugo Bastolla, Pablo Chacón

**Affiliations:** † Department of Biological Physical Chemistry, Rocasolano Institute of Physical Chemistry, CSIC, Serrano 119, 28006 Madrid, Spain; ‡ Centro de Biología Molecular “Severo Ochoa,” CSIC-UAM, Cantoblanco, 28049 Madrid, Spain

## Abstract

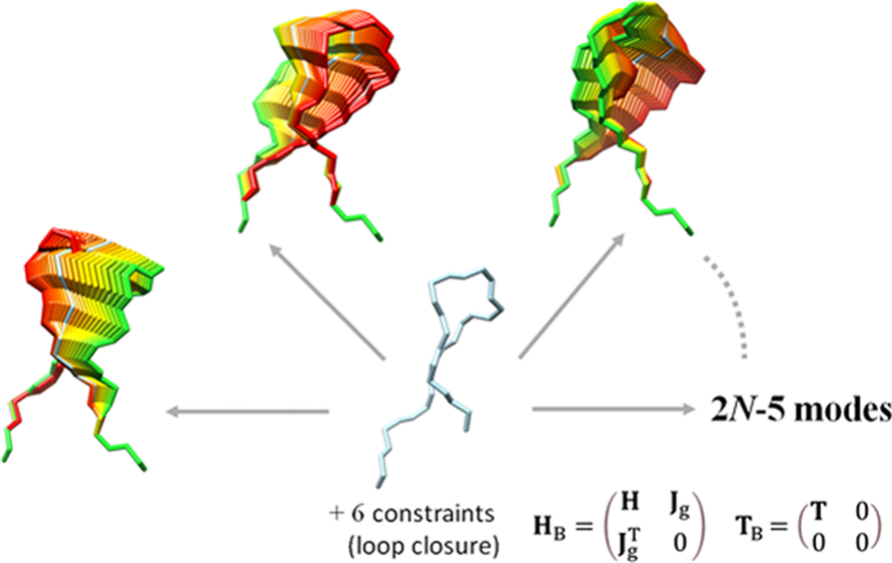

We propose and validate a novel method to efficiently explore local protein loop conformations based on a new formalism for constrained normal mode analysis (NMA) in internal coordinates. The manifold of possible loop configurations imposed by the position and orientation of the fixed loop ends is reduced to an orthogonal set of motions (or modes) encoding concerted rotations of all the backbone dihedral angles. We validate the sampling power on a set of protein loops with highly variable experimental structures and demonstrate that our approach can efficiently explore the conformational space of closed loops. We also show an acceptable resemblance of the ensembles around equilibrium conformations generated by long molecular simulations and constrained NMA on a set of exposed and diverse loops. In comparison with other methods, the main advantage is the lack of restrictions on the number of dihedrals that can be altered simultaneously. Furthermore, the method is computationally efficient since it only requires the diagonalization of a tiny matrix, and the modes of motions are energetically contextualized by the elastic network model, which includes both the loop and the neighboring residues.

## 1 Introduction

The structures of protein loops are critical for understanding mechanisms in molecular recognition, signal transduction, or enzymatic reaction. Loops can access a broad range of conformations, which makes them relatively hard to characterize at an atomic level and particularly challenging for computational prediction or design. Deep learning (DL) methods such as AlphaFold2^1^ and RoseTTAFold^2^ have dramatically impacted the protein structure prediction field. AlphaFold2 predicted the structures of many challenging protein targets near experimental resolution; however, flexible regions including flexible loops remain problematic. For example, the predicted local model quality score of AlphaFold2 negatively correlates with main-chain flexibility.^250^ Nevertheless, there are promising works for modeling antibody complementarity determining region loops.^3,4^ In addition to emerging DL approaches, template-based, ab initio, or a mix of both methods can predict stable conformations of relatively short loops (up to 10–12 residues).^5^ However, accurately sampling the great diversity of conformations of larger loops and the transitions between them is still a computational challenge.

The main ingredient for loop modeling and loop refinement tools, along with scoring, is the sampling method that must satisfy the closure of the loop. Among diverse methodologies,^6^ inverse kinematics formulations are a popular alternative for loop sampling that is either based on analytical solutions or numerical optimization techniques. The main analytical technique^7−9^ yields directly closed conformations of a given tripeptide loop (or any six torsion angles) by solving a 16th-degree polynomial. Larger loops are, in essence, solved by the iterative application of this polynomial resultant method to three large subfragments. Numerical methods are conceptually simpler and can be directly applied to long loops. The cyclic coordinate descent (CCD) method superimposes mobile and target loop anchors by finding the optimal dihedral angle for the current rotation bond.^10^ Similar to CCD, one of us developed random coordinate descent (RCD),^11^ which includes geometric filters and uses spinor matrices to yield a more efficient conformational sampling. Random tweak,^12,13^ systematic conformational search,^14,15^ bond scaling,^16−18^ Monte Carlo,^18−20^ and hashing^21^ are also relevant approaches. Despite the success of such sampling algorithms,^6^ improving their efficacy and accuracy for long loops remains challenging since the number of possible conformations increases exponentially. Alternative or complementary approaches are therefore needed to enhance the performance and sampling power.

Normal mode analysis (NMA) has become increasingly popular to predict macromolecular dynamics, from small proteins to large assemblies, since it yields a reasonable description of experimentally observed functional motions at low computational cost.^22−24^ NMA is widely used to efficiently explore collective motions in many challenging problems such as docking^25−29^ or structural fitting to experimental data such as electron microscopy density maps.^30−32^ In the context of loop modeling, successful applications of NMA include, for example, the generation of alternative loop receptor conformations in cAMP-dependent protein kinase.^33^ However, although NMA provides an efficient and rather inexpensive description of the macromolecular conformational space, it is less directly suitable to generate loop conformations because it does not guarantee loop closure. To reconcile normal modes with concerted local motions, it was necessary to bias the sampling by drastically reducing the number of modes, including additional constraints, and/or actively repairing the covalent structure at the end of the loop.

In this paper, we describe a new formalism for constrained NMA that allows representing the manifold of closed-loop configurations as a combination of normal modes. Perturbing the loop structure with any combination of such modes directly generates alternative conformations that fulfill loop closure. Unlike other methods that generate concerted motions in loops, our approach is not limited to modifying a few dihedrals, but it considers all dihedrals simultaneously. It is very efficient; it only requires the diagonalization of a tiny matrix. Moreover, since the elastic network considers both the loop and neighboring residues, the loop closure problem remains grounded in the context of the whole protein. These unique features, as we describe below, allow us to efficiently explore the accessible loop conformations in a new and promising way.

## 2 Methods

### 2.1 NMA in Internal Coordinates

The details of our NMA framework in internal coordinates were described previously^34−36^ and are similar to those implemented in ref (37). Briefly, the internal mobile coordinates are defined by the canonical backbone dihedral angles, while the remaining dihedral angles and all covalent bond lengths and angles are fixed. The potential energy is approximated by an elastic network of harmonic oscillators connecting the heavy atoms and vibrating around the equilibrium conformation represented in the PDB file. The corresponding vibrational displacements are directly computed from the Lagrangian equations of motion by solving the generalized eigenvalue problem in internal coordinates^38^
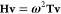
1where **H** is the Hessian matrix or the second derivatives of the potential energy, **T** represents the kinetic energy matrix, **v** = (***v***_1_,***v***_2_,...,***v***_*n*_) is the eigenvector matrix in the space of the *n* internal coordinates of the system **q** = (*q*_1_,*q*_2_,...,*q_n_*), and **ω**is the diagonal matrix of the eigenvalues (squared oscillation frequency). For simplicity, we do not implement the Eckart conditions, which impose that the kinetic energy does not contain any rigid body motion. This is not necessary here, since our goal consists of sampling the loop conformations, which is achieved since the normal modes constitute a complete system even without the Eckart conditions.

### 2.2 Constrained NMA

Even though analytical solutions to the equations of motion extracted from the whole protein structure can describe loop motions, the application of NMA to a loop does not guarantee its closure. We aim to determine the normal modes in such a way that their application from an initial anchor *N*-terminal guarantees that the *C*-terminal end remains properly connected and oriented. For this purpose, we implement an NMA variant that enforces *C* geometrical constraints *g*_1_, *g*_2_, ..., *g*_C_.

In general, the eigensystem of a matrix can be formulated as the minimization of the quadratic form of such a matrix in the vector space (***v***_1_, ***v***_2_, ..., ***v***_*n*_) subject to the normalization constraint ∑_*a* = 1_^*n*^***v***_*a*_^2^ = 1 imposed through a Lagrange multiplier that coincides with the eigenvalue (subsequent eigenvectors can be regarded as minimization problems in the orthogonal subspaces). In the same way, constrained normal modes can be formulated as a constrained minimization problem with Lagrange multipliers λ_*k*_ associated to each constraint *g_k_*. The resulting equations are
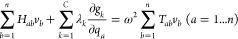

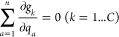
2

In these *n* + *C* equations, the eigenvector components *v_b_*, the Lagrange multipliers λ*_k_,* and the eigenvalue ω^2^ must be determined self-consistently. Therefore, we define the extended generalized eigenvector problem **H**_B_***v*** = **ω**^2^**T**_B_***v*** with eigenvector components (*v*_1_, *v*_2_, ..., *v_n_*, λ_1_, λ_2_, ..., λ_*C*_) and extended matrices
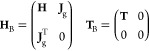
3

Here, **J**_g_ denotes the Jacobian matrix of the *C* constraints
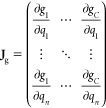
4

**H**_B_ is called the bordered Hessian. It involves the second-order derivatives of the Lagrange function, defined as the sum of the potential energy and the Lagrange multipliers for the *n* + *C* extended coordinates. It is easy to see that the modes of this extended eigensystem coincide with the solutions of the constrained eigensystem (eq 2). Note that this formulation is general: a constrained eigenvector problem can be posed as an extended generalized eigenvector problem.

The extended kinetic energy **T**_B_ has *C* zero components corresponding to the λ_*ak*_, and the system has *C* additional null modes corresponding to the constraints. Therefore, the extended eigensystem has *n* + *C* – 2*C* = *n* – *C* useful eigenvectors corresponding to the constrained normal modes of the loop. Note that the normal modes produced by this method (**v**_B_) are orthogonal in the *n* + *C* dimensional space of internal degrees of freedom plus constraints, but not necessarily in the *n*-dimensional space of internal degrees of freedom.

The normal modes generated by this formalism encode motions that exactly respect the selected constraints but only near the initial conformation. This is an inherent limitation of the linear approximation between internal and Cartesian coordinates. Therefore, to avoid potential geometrical distortions at larger excursions from the initial structure (e.g., shifts at the *C*-terminal end), the angular motions are limited to 1° at most and NMA calculations are updated iteratively upon every move. The new modes are matched to the previous ones using their dot product. This remains computationally efficient since each step only requires the computation and diagonalization of a very small matrix.

### 2.3 Loop Closure Constraints

We consider a loop consisting of *N* flexible residues and terminating with a rigid carboxy-terminal (*C*_t_) anchor residue. We take as degrees of freedom the φ and ψ angles of the *N* flexible residues plus the φ angle of *C*_t_, that is, *n* = *2N* +1. To maintain the loop closed with the proper orientation, it is sufficient to fix the Cartesian coordinates of three atoms, for example, the N, C_α_, and C atoms of *C*_t_, with respect to the combined effect of motions along the *n* internal coordinates *q_a_*. In practice, only *C* = *6* constraints are necessary, since the N–C_α_ and C_α_–C bond lengths and the N–C_α_–C angle are fixed. For a loop of *N* flexible residues, the number of effective modes of motion is thus equal to *n* – *C* = 2*N* – 5. We choose the first three constraints as the Cartesian coordinates of the C_α_ atom in *C*_t_
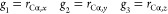
5

The other three constraints fix the coordinates of the N and C atoms of *C*_t_, using a reference frame built around the C_α_ atom: , , and *w⃗* = *u⃗* × *v⃗*.
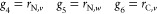
6

The fixed N–C_α_ and C_α_–C bond lengths and the N–C_α_–C angle preclude explicitly adding more constraints (e.g., *r*_N,*u,*_*r*_C,*u,*_ and *r*_C,*w*_).

### 2.4 Benchmark Loop Data Sets

Our approach is validated on a set of highly variable loops observed in multiple stable conformations and compiled by others.^39^ This set includes 30 loops that are 10–15 residues long. Each loop case is associated with an ensemble of 2–11 different conformations. The complete data set _A_ includes 392 possible transitions considering each pair of structures of the same loop, both in the forward and backward direction. After superposition of the full initial and final structures using KPAX,^40^ the backbone RMSD between initial and target conformations of the loop ranges from 0.1 to 11.3 Å, with an average of 2.5 Å. We also consider the subset _C_ of the 184 most challenging cases, where the initial and final loop conformations differ by more than 2 Å. This set is further divided into _CS_ and _CL_, which contain 80 challenging conformational transitions for shorter loops (10–12 residues) and 104 for longer loops (13–15 residues).

A second benchmark set consists of 15 exposed and diverse loops employed to test loop predictions using replica exchange molecular simulations (REMD) with RSFF2C force field.^41^ These loops had a resolution of <2.0 Å, Rfactor < 0.3, sequence identity <20%, and an average B-factor < 35. We extend the length of the loops by one residue at both ends to minimize the deviations of the anchors found in the REMD simulations. The length of the loops ranges from 12 to 18 residues. The initial MD structures were prepared via implicit MD simulations at high temperatures to guarantee that they are far away from the crystallographic ones (>10 Å). Trajectories and initial REMD structures and the corresponding simulations were kindly provided by the authors, with structures already superimposed by the anchors.

### 2.5 Comparison with Molecular Dynamics Simulations

Besides evaluating the ability of our approach to describe the transitions between pairs of loop conformations, we used the REMD simulation data to compare the conformational ensembles generated by our NMA in torsion angles with MD equilibrium ensembles. In practice, we focus on the last μs of these 5 μs long REMD trajectories. These trajectories include rigid loops such as 2ns0, 4dpb, 3bv8, and 5k2l with RMSD < 1 Å over the last μs simulation and more flexible loops such as 5e9p, 3k3v, and 3dkm with deviations ∼2–4 Å.

For each of the 15 loops, the NMA input structure was chosen as the backbone structure closest to the MD ensemble average, which belongs to the most populated conformations and is typically close to the crystallographic structure. The NMA ensemble is created by first generating a random combination of modes that define a target direction. The conformation of the loop is iteratively flexed in this direction until reaching a maximum amplitude, chosen to have similar RMSD deviations to the REMD simulations (1.5 Å for the rigid loops and 2–4 Å for the flexible ones). This process is repeated until 10,000 loops are generated, and we save the loop coordinates when they deviate more than 0.1 Å RMSD.

These NMA “pseudo-trajectories” around equilibrium are compared with the REMD trajectories through principal component analysis (PCA), that is, we diagonalize the Cartesian covariance matrix of the correlated fluctuations of pairs of backbone atoms about the closest to average loop structure to obtain a set of eigenvectors and eigenvalues. The eigenvectors describe collective directions and the eigenvalues represent the amount of variance explained by each eigenvector. For each loop, we compute the overlap between the spaces spanned by the *m* most relevant eigenvectors of the REMD and the *m* corresponding NMA eigenvectors  as^42,43^
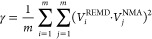
7

Here, the eigenvectors are ranked according to their contribution to the structural variance. The overlap is one if all eigenvectors and all degrees of freedom are used; however, we limit the sum to the smallest number of eigenvectors needed to explain 90% of the variance in each respective ensemble. Similar results hold if we consider a fixed number of modes (e.g., 10 or 20 first modes; see Table S1). We also compute Z-scores in order to refer the gamma indexes to a background model
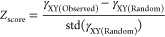
8where the random models were obtained by diagonalizing a pseudo-covariance matrix obtained by random permutation of the backbone atoms for each snapshot, and the standard deviations were obtained by considering 1000 different random models.

## 3 Results

### 3.1 Illustrative Example

**Figure 1:**
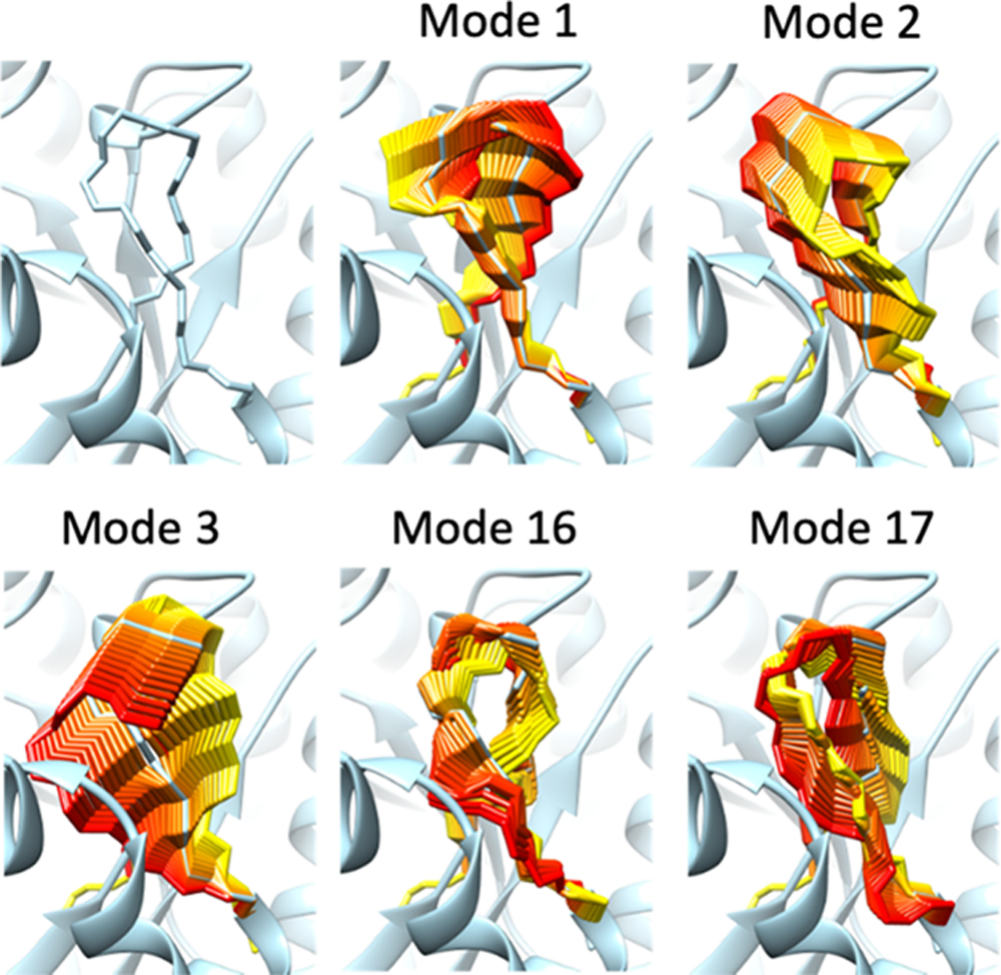
Constrained normal modes of the 66–76 loop in the structure of the *Bordetella bronchiseptica* hydrolase (PDB: 3IRS, chain C). The conformational ensembles (rainbow colored) represent the motions encoded in each mode. They are generated by perturbing the loop conformation along a given mode direction until reaching an RMSD of 3 Å from the initial loop structure (light blue backbone stick representation). The displayed ensembles include intermediate conformations, every 0.25 Å along the pathway. The rainbow colors indicate the amplitude and direction, from positive (yellow) to negative (red). Only the 3 lowest and the 2 highest frequency modes are displayed here, but the motions along all 17 modes are visualized in Movie S1.

To illustrate our proposed formalism, we performed constrained NMA on a loop of *N* = 11 residues, using the φ and ψ dihedral angles as variables. The motions of the loop, along the directions defined by each of the 2*N* – 5 = 17 constrained modes, are visualized in Figure 1 and Movie S1. It is apparent that these modes encode concerted rotations around the backbone dihedral angles, such that the stereochemistry at loop ends is preserved. As with unconstrained NMA, low-frequency modes (e.g., modes 1–3) tend to correspond to more collective motions, whereas the high frequency modes (e.g., modes 16–17) are more local. Although we illustrate here only single modes, perturbing the loop structure with any combination of modes also generates valid closed-loop conformations.

It is important to emphasize that the motions sampled by our method do not have to arbitrarily select driving torsions, in contrast with some popular approaches,^7−9^ which are typically limited to modifying six dihedrals simultaneously.

### 3.2 Validation of the Closed-Loop Modal Space

To verify that the motions encoded in our local NMA modes provide sufficient coverage of the closed-loop conformational space, we evaluate the ability of our model to reproduce 392 structural transitions between experimentally observed stable loop conformations (see Section 2). Starting from the initial structure, the conformation of the loop is progressively flexed toward the target structure using only the motions encoded in the constrained modes. At each step, we compute the modes and linearly combine them according to their overlap with the target direction, that is, the vector between initial and target structures. The process is repeated iteratively until convergence.

Final RMSD obtained between the flexed and the target loop conformations against the initial RMSD for all 392 transitions are shown in Table 1 and Figure S1. In the full data set _A_, the average RMSD drops from 2.5 to 0.55 Å (Table 1). Note that we cannot reach complete convergence because our internal normal modes do not modify bond angles, bond lengths, and ω torsion angles (see ref (44)). There is some correlation between initial and final RMSD (Figure S1), but our procedure remains successful even for the most challenging cases. Indeed, in the subset _C_ that only includes large-amplitude transitions, the average RMSD of 4.2 Å is reduced to 0.76 Å. Interestingly, even though the conformational space increases exponentially with the length of the loop, the average final RMSD is slightly smaller (0.69 vs 0.85 Å) for the longer loops (_CL_, size 13–15) than for shorter loops (_CS_, size 10–12). This nicely illustrates one of the main advantages of our methodology, which lies in its ability to explore the conformational space by altering all dihedrals simultaneously in a concerted manner even with long loops.

**Table 1:** Mean Values of the Initial and Final Morphing RMSD

data set	*N*_pairs_	RMSD^a^_initial_	RMSD^b^_final_	RMSD^c^_final_ (flanks aligned)	RMSD^d^_final_ (incl. ω)
_A_	392	2.5 ± 2.5 Å	0.55 ± 0.33 Å	0.51 ± 0.31 Å	0.44 ± 0.31 Å
_C_	184	4.2 ± 2.2 Å	0.76 ± 0.28 Å	0.69 ± 0.27 Å	0.63 ± 0.27 Å
_CS_	80	4.3 ± 2.1 Å	0.85 ± 0.29 Å	0.73 ± 0.26 Å	0.71 ± 0.31 Å
_CL_	104	4.2 ± 2.3 Å	0.69 ± 0.27 Å	0.65 ± 0.26 Å	0.58 ± 0.23 Å
REMD	15	10.4 ± 3.3 Å	0.71 ± 0.18 Å	0.71 ± 0.17 Å	0.64 ± 0.25 Å

a Mean and standard deviation of the initial RMSD between pairs of experimentally observed loop conformations, on the full dataset and three subsets (see Section 2).

b Mean and standard deviation of the final RMSD between target and reconstructed loop conformation.

c The procedure is performed after the superposition of the loop-flanking residues, rather than the complete structures. The flanking residues are the anchors and two more on either side of the loop.

d The ω backbone dihedrals are considered as degrees of freedom, along φ and ψ.

**Figure 2:**
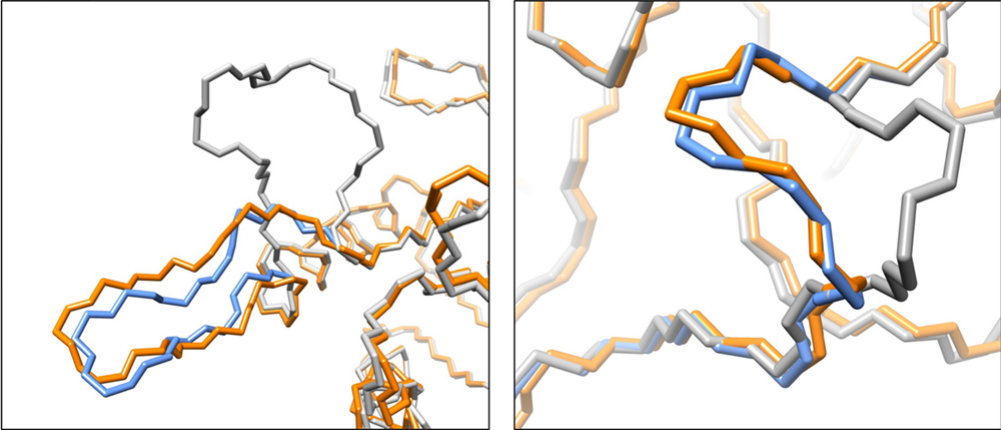
Two illustrative morphing cases: (1) on the left: transition of loops 81–93 of the PPPK kinase, from 3hsz (gray) to 3ht0 (orange); (2) on the right: transition of loops 317–337 of the MopE protein, from 2vov (gray) to 2vox (orange). The corresponding final morphed conformations are represented in blue.

Two individual examples are given in Figure 2 and Videos S2 and S3. In the most extreme case, the modal displacements are sufficient to capture the large 11.3 Å backbone RMSD conformational change displayed by a loop in PPPK kinase. Although this is one of the largest amplitude transitions in our data set, the difference between target and final conformations is barely 1.2 Å. In the other example, the loop conformations in two structures of protein MopE originally present an RMSD of 3 Å, and this deviation is reduced to 0.5 Å after the application of our morphing procedure.

Overall, these results demonstrate the ability of the modal space derived from our formalism to describe transitions between alternative closed-loop conformations, even in the case of longer loops and/or large-amplitude conformational changes. It should be noted that some unavoidable factors preclude an exact match between the modeled and target conformations. First, the structural superimposition of the loop anchors in the two experimental conformations is not perfect. In fact, the loop-flanking residues (three on each side of the loop) present an average RMSD of 0.7 Å after the superposition of the two structures. This imposes a background bias impossible to recover since only the loop residues are free to move. We repeated our procedure after the superposition of only the loop-flanking residues rather than the complete structures. The average RMSD for the loop-flanking residues is then reduced to 0.3 Å, but the results for the loops themselves are only slightly improved (Table 1). Another, probably more critical, source of discrepancy lies in the fact that only the φ and ψ dihedral angles are considered as degrees of freedom. The other internal coordinates (bond lengths, valence angles, and ω dihedrals) are fully rigid, even though they often take slightly different values between the pairs of experimental conformations. As shown previously, such small differences in internal coordinates create deviations that quickly propagate and can have a major impact on the reconstructed Cartesian coordinates, even on the scale of small peptides or loops.^44^ Including the ω dihedral angles in the set of degrees of freedom considered by our procedure does indeed slightly improve the performances, with a final RMSD of 0.44 Å, down from 0.55 Å when only φ–ψ are considered (Table 1).

Furthermore, we examined a set of large conformation changes of loops whose initial structure was generated by perturbing the loop in the crystallographic structure through MD simulations at high temperature.^41^ We carried out a similar morphing experiment to test how our NMA approach covers the closed-loop conformational space in these transitions of extreme amplitude, and we found that the backbone RMSD between initial and target structures drops from 10.42 to 0.64 Å (Table 1).

### 3.3 Comparison with Molecular Dynamics

The REMD simulation data were used to evaluate the correspondence between the conformational space generated by our approach and MD equilibrium ensembles (see Section 2). Because of its high computational cost, the application of atomistic MD simulations is often limited to a post-processing stage to refine loop solutions provided by much faster loop sampling methods. Our NMA in torsion angle space is much faster, but is limited with respect to MD simulations: (1) Only the loop φ–ψ torsion angles are modified, while all other internal degrees of freedom are fixed. Therefore, it does not include potential conformational changes of other parts of the protein. (2) Our approach does not consider side chains, neither as degrees of freedom nor as interacting atoms. (3) Our normal modes are based on a structure-based energy function (elastic network model) that places the initial structure at the minimum of the energy. In contrast, the physics-based energy function adopted in MD/REMD can be used to also score and predict a most stable loop structure. (4) The harmonic approximation is applied, which is not accurate for large displacements. Since normal modes constitute a complete system, (3) and (4) do not restrict their ability to reproduce any possible displacement in the space of torsion angles. However, if we only consider a limited set of low-frequency normal modes in order to increase sampling efficiency, the approximations of the energy function may limit the results.

Therefore, we compute the overlap γ between the most relevant PCA eigenvectors from the NMA and MD conformational ensembles, that is, we consider the smallest number of eigenvectors that can explain 90% of the variance in each conformational space. The summary of comparative results is shown in Table 2 and detailed in Table S1. Around 5–6 modes are needed to explain 90% of the variance of the NMA ensemble, whereas for the MD ensemble, this number varies from 2 to 14. Even though the NMA only considers φ and ψ torsion angles while the MD moves all Cartesian coordinates, the overlap is equal to 0.7 on average, indicating an imperfect but good similarity between the two ensembles. The large Z-score values (>100 in most cases) confirm that such overlaps are extremely unlikely to occur randomly.

**Table 2:** REMD and NMA Sampling Space Comparation

	RMSD^a^		modes^b^		90%var^c^		Bf^d^	RMSD^e^ cryst.			NMA^f^	
	MD	NMA	MD	NMA	γ_90%_	Z_90%_	*s*	avg	NMA	MD	time	speed
2eaq	1.5	1.4	2	8	0.64	602	0.85	1.51	1.15	0.99	37.8	1.2
5w0g	1.0	1.3	6	5	0.69	184	0.56	0.75	0.48	0.32	27.2	1.6
2ns0	0.7	1.3	9	6	0.69	196	0.90	0.57	0.44	0.32	28.6	1.2
4dpb	0.8	1.3	7	6	0.73	271	0.75	0.50	0.42	0.27	30.5	2.0
5nod	1.0	1.4	5	4	0.80	183	0.92	1.18	0.47	0.40	26.1	1.5
6elm	1.6	1.3	7	6	0.70	559	0.93	1.49	0.96	0.48	32.2	1.7
3bv8	0.5	1.3	14	5	0.78	111	0.97	0.30	0.26	0.25	31.8	1.5
5e9p	2.8	2.3	3	5	0.71	47	0.98	2.34	1.08	0.31	31.7	1.8
4bpf	1.3	1.3	4	5	0.45	48	0.90	0.57	0.44	0.26	35.2	1.5
6fmb	1.2	1.3	8	6	0.74	116	0.89	0.99	0.55	0.41	41.3	0.9
5k2l	0.8	1.3	10	5	0.74	279	0.93	0.55	0.33	0.21	31.7	1.3
3k3v	3.5	2.4	5	5	0.67	38	0.97	1.74	1.32	0.84	36.7	0.9
3fdr	1.1	1.1	7	9	0.80	231	0.78	0.62	0.48	0.45	55.0	3.1
4qy7	1.3	1.2	5	5	0.65	144	0.60	0.81	0.69	0.46	37.3	5.0
3dkm	3.6	3.4	4	3	0.73	107	0.89	3.63	2.46	2.40	39.8	1.7
Avg	1.5	1.5	6.4	5.5	0.70	207	0.85	1.13	0.78	0.56	34.8	1.8

a Backbone RMSD deviation from the average reference loop sampled by REMD.

b Number of eigenvectors needed to explain 90% of the variance.

c Corresponding similarity indexes and Z-scores obtained with 90% variance.

d B-factor Spearman correlations.

e Minimum of RMSD deviation with respect to the crystal conformation.

f Required time for the sampling 10K loops, and speed factor with respect to RCD sampling.

In addition, the B-factor profiles derived from NMA and REMD eigenvectors correlate well, indicating a similarly good correspondence at the residue level (Tables 2 and S1 and Figure S2). The Spearman’s correlation coefficients are between 0.76 and 0.98, except in two cases: the highly localized flexibility at the *C*-terminal loop of 4qy7 and the larger flexibility at the *N*-terminal (*N*_t_) end of 5w0g observed in the atomistic simulations are not captured by our NMA. A possible explanation for the former case is related to the fact that the high flexibility is localized at the end of a region with high helix propensity (fraction 0.37, sequence DDLLKR). The explanation for the latter case is that the *N*_t_ anchor is quite mobile, as shown in Figure 4 of the report by Feng et al.^41^

In Table 2, we also show the RMSD from the original crystal structure. The NMA approach reaches configurations almost as close to the crystal as the best match obtained during the REMD, although bond angles and lengths are fixed and a detailed energy model is not considered. Admittedly, the starting structures are already close to the targets (0.99 Å on average), so the improvements are necessarily small. We observed an improvement over 1 Å in two cases: 5e9p and 3dkm, where the initial average loop is more than 2 Å away. In the first case, the REMD simulation reaches the crystal conformation (0.31 Å) and NMA is close (1.08 Å). For 3dkm, the loop with the largest initial average RMSD (3.63 Å), both REMD and NMA are still 2.40 and 2.46 Å away.

### 3.4 Computational Efficiency

Besides the potential limitation with large and flexible loops, the major drawback of MD-based loop sampling is the computational cost. Interestingly, the combination of NMA in internal coordinates with REMD and other MD sampling strategies has been successfully employed to drastically reduce this computational cost.^45,46^

Our NMA approach is fast. It only takes on average 35 s to generate and save 10k closed conformations for the loops included in the REMD benchmark, that is, less than 4 ms per loop on a Linux box with an I7-6770HQ processor. In Table 2, we compare the run time of our algorithm with RCD,^11^ one of the fastest methods to generate an ensemble of backbone closed loops, also developed by us. The NMA is on the overage 1.8 times faster than RCD in generating the same number of closed loops. Note that these sampling algorithms are quite distinct in nature: RCD samples the geometrically feasible space of closed loops in a stochastic manner rather than exploring the space around a closed loop exhaustively. The faster NMA approach also yields an improved agreement with MD ensembles, with an average overlap γ = 0.70 vs 0.66 for RCD and average B-factor correlation of 0.76 vs 0.71 for RCD (Tables S1 and S2). Note that the overlap calculated between the last and the previous last μs of the simulation had much higher values of 0.9 (Table S3), as they correspond to an almost “perfect” match.

## 4 Discussion

We introduced here a novel and simple formalism to generate alternative closed-loop conformations by perturbing an initial structure with constrained modes. These modes naturally encode concerted motions of all the dihedral angles that keep the loop properly closed. Moreover, it does remain computationally efficient, since the major burden is the solution of an eigenvalue problem of size 2*N* – 5, where *N* is the number of flexible residues.

We showed an acceptable resemblance of the ensembles around equilibrium conformations generated by long REMD simulations and constrained NMA on a set of exposed and diverse loops. Despite all the approximations of our NMA approach with respect to more accurate MD simulations (Section 3.3), this successful correspondence suggests that the constraints at the ends and the neighboring residues largely limit the conformational space, and also that loops behave more harmonically than expected. Using the REMD data set and other experimentally observed highly variable loop conformations, we ensured that our method is able to reproduce closed-loop structural transitions with high precision and to comprehensively explore the accessible conformational space of any loop. It is worth noting that such interpolations between structures, with a simple RMSD-based step descent procedure, do not constitute the ultimate objective of the proposed methodology. The most interesting and useful feature of our new formalism is the potential to efficiently explore the possible motions of a flexible loop around a given equilibrium structure, which can be ranked using more elaborate and detailed energy functions. Although our approach does not provide an energetic evaluation or scoring, it is conceptually simple and relatively easy to implement, and it can readily be incorporated into current loop refinement/modeling scoring protocols, or even merged with REMD and other MD sampling strategies.^45,46^ To this end, source code, Linux binaries, all the test sets, and the corresponding results are fully available. Another advantage of our approach is that the elastic network model includes both the loop and the neighboring residues. Fully contextualized, albeit coarse-grained, energetic and clash-avoidance considerations are therefore implicitly embedded within the computed modes.

In order to further progress from this innovative formalism toward a fully fledged loop modeling package, we plan to include it in our loop modeling RCD+ server as an alternative sampling strategy.^47^ The combination of both approaches could yield a more effective search strategy by exploiting the stochasticity of RCD and the local exhaustiveness of the NMA approach. In principle, this method is not limited to short loops and could be applied to sample flexible protein regions of any size. However, more research is needed to apply it to larger protein segments motions. We also aim to combine it with regularized linear fitting approaches^44,48^ for fine-tuned sampling and to alleviate the impact of small variations in *C*-terminal bond length and valence angles.

## Supporting Information Available

Final RMSD between target and reconstructed loop conformations (Figure S1); calculated B-factors for the REMD simulation, NMA, and RCD samplings (Figure S2); comparative measures of the REMD and NMA ensembles (Table S1); comparative measures of REMD and RCD ensembles (Table S2); comparative measures of the last and the previous last nanosecond of the REMD simulation trajectories (Table S3); RMSDs of the initial REMD loop conformation and final morphed conformation obtained with the NMA (Table S4) (PDF)
**Video S1.** Constrained NMA modal motions of the 11 residues-long loop (residues from 66 to 76) from the hydrolase structure of *Bordetella bronchiseptica* (PDB: 3ris). The motions of the 17 modes are displayed sequentially with different color stick representations (MOV)
**Video S2.** PPPK kinase transition pathway of the loop 81-93 from 3hsz (gray) to 3ht0 (orange) structures (MOV)
**Video S3.** MopE protein transition pathway of the loop 317-327 from 2vov (gray) to 2vox (orange) structures (MOV)

## Supplementary Material

ci2c00870_si_001.pdf

ci2c00870_si_002.mov

ci2c00870_si_003.mov

ci2c00870_si_004.mov

## Notes

The source code, binaries, and all the test data and the corresponding results are freely available from github (https://github.com/chaconlab/ilmode) and from https://chaconlab.org/multiscale-simulations/ilmode.
