# Supplementary material for: Local Normal Mode Analysis for Fast Loop Conformational Sampling
Source: J Chem Inf Model. 2022 Sep 13;62(18):4561–8. doi: 10.1021/acs.jcim.2c00870 (PMC9516680; doi:10.1021/acs.jcim.2c00870)
Supplement: Supplementary file 1 — ci2c00870_si_001.pdf [file ci2c00870_si_001.pdf]

Supporting Information

Local Normal Mode Analysis for Fast Loop Conformational Sampling

José Ramón López-Blanco[1], Yves Dehouck[2], Ugo Bastolla[2], and Pablo Chacón[1]*

[1]Department of Biological Physical Chemistry, Rocasolano Institute of Physical Chemistry. CSIC., Serrano 119, 28006 Madrid, Spain

[2]Centro de Biología Molecular "Severo Ochoa," CSIC-UAM Cantoblanco, 28049 Madrid, Spain.

Corresponding Author: pablo@chaconlab.org

Video S1. Constrained NMA modal motions of the 11 residues-long loop (residues from 66 to 76) from the hydrolase structure of *Bordetella bronchiseptica* (PDB: 3ris). The motions of the 17 modes are displayed sequentially with different color stick representations. For details see the caption of Figure 1.

Video S2. PPPK kinase transition pathway of the loop 81-93 from 3hsz (grey) to 3ht0 (orange) structures.

Video S3. MopE protein transition pathway of the loop 317-327 from 2vov (grey) to 2vox (orange) structures.

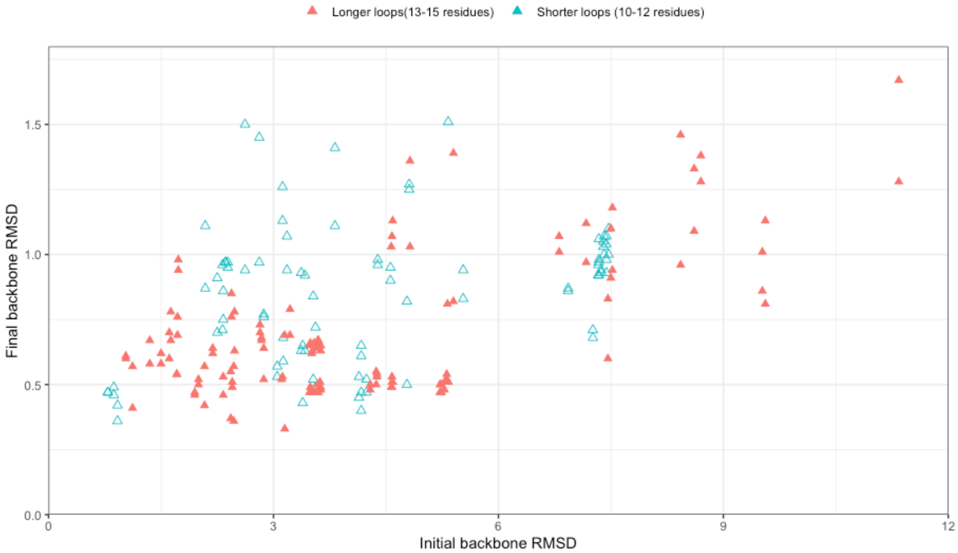

Figure S1. Final RMSD between target and reconstructed loop conformations, as a function of the initial RMSD between the two experimentally observed loop conformations in the challenging transitions subset (Initial RMSD >2 Å). Shorter (10-12 residues) and longer (13-15 residues) loops are represented distinctly. The Pearson correlation coefficient between initial and final RMSD is 0.74 ($p<10^{-15}$) over the whole set, and 0.50 ($p<10^{-10}$) over challenging transitions only.

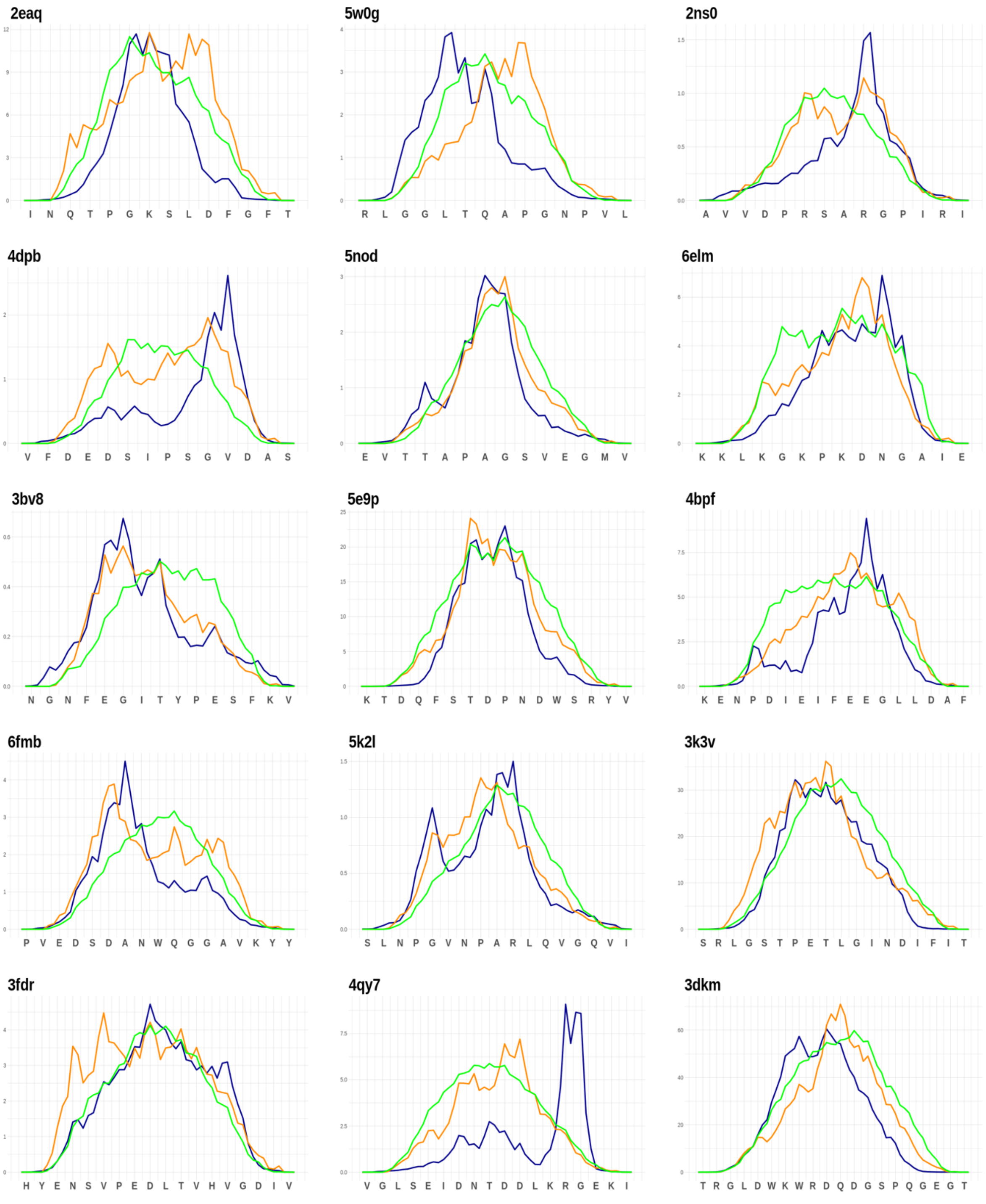

Figure S2. Calculated B-factors for the REMD simulation (blue), NMA (orange) and RCD (green) samplings. The NMA and RCD B-factors were fitted to the corresponding REMD simulation. The corresponding Pearson and Spearman correlations (excluding the anchors) are in Table S1-2.

Table S1. Comparative measures of the REMD and NMA ensembles.

	[a]RMSD REMD		[b]RMSD NMA		90% variance				[e]10 first modes			[e]20 first modes			[f]B-Factor		[g]Sampling NMA	
**PDB**	Avg	σ	Avg	σ	[c]REMD	[c]NMA	[d]$\gamma_{90\%}$	[d]$Z_{90\%}$	γ_{10}	Z_{10}	var	γ_{20}	Z_{20}	var	PCC	Spearman	time Sec	Speed factor
**2eaq**	1.52	1.29	1.45	0.86	2	8	0.64	602	0.69	90	0.98	0.77	213	0.99	0.73	0.85	37.8	1.2
**5w0g**	1.03	0.62	1.35	0.86	6	5	0.69	184	0.69	164	0.94	0.76	222	0.98	0.32	0.56	27.2	1.6
**2ns0**	0.66	0.22	1.35	0.84	9	6	0.69	196	0.69	196	0.91	0.75	137	0.96	0.80	0.9	28.6	1.2
**4dpb**	0.79	0.40	1.32	0.88	7	6	0.73	271	0.77	263	0.93	0.78	248	0.97	0.62	0.75	30.5	2.0
**5nod**	1.04	0.38	1.42	0.87	5	4	0.80	183	0.82	157	0.95	0.82	313	0.98	0.95	0.92	26.1	1.5
**6elm**	1.57	0.86	1.35	0.85	7	6	0.70	559	0.76	518	0.94	0.78	215	0.98	0.89	0.93	32.2	1.7
**3bv8**	0.52	0.16	1.34	0.86	14	5	0.78	111	0.76	579	0.84	0.73	346	0.94	0.94	0.97	31.8	1.5
**5e9p**	2.85	0.90	2.29	1.64	3	5	0.71	47	0.67	240	0.98	0.74	265	0.99	0.97	0.98	31.7	1.8
**4bpf**	1.27	1.01	1.34	0.85	4	5	0.45	48	0.61	77	0.96	0.73	35	0.99	0.84	0.9	35.2	1.5
**6fmb**	1.08	0.54	1.34	0.85	8	6	0.74	116	0.75	136	0.91	0.75	185	0.97	0.83	0.89	41.3	0.9
**5k2l**	0.77	0.29	1.34	0.86	10	5	0.74	279	0.75	189	0.89	0.78	81	0.96	0.87	0.93	30.4	1.3
**3k3v**	3.54	1.69	2.35	2.18	5	5	0.67	38	0.70	43	0.96	0.72	48	0.99	0.94	0.97	36.7	0.9
**3fdr**	1.13	1.06	1.13	0.84	7	9	0.80	231	0.78	218	0.94	0.77	71	0.98	0.85	0.78	55.0	3.1
**4qy7**	1.32	0.77	1.25	0.86	5	5	0.65	144	0.77	315	0.95	0.77	120	0.98	0.07	0.6	37.3	5.0
**3dkm**	3.57	3.61	3.43	2.47	4	3	0.73	107	0.68	83	0.97	0.73	39	0.99	0.85	0.89	39.8	1.7
	1.51	0.92	1.52	1.02	6	5	0.70	207	0.72	217	0.94	0.76	171	0.98	0.76	0.85	34.8	1.8

[a]RMSD deviation of the REMD simulation. [b]RMSD deviation of NMA ensemble. RMSD deviations consider using only the backbone atoms and are computed respect to the closest loop to the average structure sampled by REMD. [c]Number of eigenvectors needed to explain 90% of the variance obtained by REMD and NMA. [d]Corresponding similarity indexes and Z-scores obtained with 90% variance. In [e] and [f], equivalent scores obtained with the first 10 and 20 modes, respectively. [f]Pearson and Spearman correlations of the B-factors obtained by REMD and NMA approaches. [g]Required time in seconds for sampling the 10000 loops with NMA, and speed factor respect RCD sampling (Table S2).

Table S2. Comparative measures of REMD and RCD ensembles.

	[a]RMSD REMD		[b]RMSD RCD		90% variance				[e]10 first modes			[e]20 first modes			[f]B-Factor		[g]Sampling RCD
PDB	Avg	σ	Avg	σ	[c]REMD	[c]RCD	[d]$\gamma_{90\%}$	[d]$Z_{90\%}$	γ_{10}	Z_{10}	var	γ_{20}	Z_{20}	var	PCC	Spearman	time Sec
**2eaq**	1.52	1.29	6	1.87	2	5	0.60	74	0.70	50	0.98	0.81	127	0.99	0.91	0.98	44.7
**5w0g**	1.03	0.62	5.86	1.83	6	5	0.71	74	0.68	117	0.94	0.80	48	0.98	0.74	0.81	43.4
**2ns0**	0.66	0.22	5.71	1.88	9	5	0.66	14	0.66	14	0.91	0.69	87	0.96	0.58	0.79	34.6
**4dpb**	0.79	0.40	6.06	2.36	7	5	0.67	23	0.73	36	0.93	0.76	54	0.97	0.19	0.49	61.8
**5nod**	1.04	0.38	7.59	3.59	5	3	0.62	20	0.70	53	0.95	0.74	74	0.98	0.86	0.90	38.5
**6elm**	1.57	0.86	3.87	1.22	7	7	0.67	82	0.71	235	0.94	0.82	133	0.98	0.84	0.88	56.1
**3bv8**	0.52	0.16	5.62	1.98	14	6	0.72	175	0.71	51	0.84	0.69	23	0.94	0.56	0.64	48.1
**5e9p**	2.85	0.90	7.81	2.16	3	6	0.70	43	0.68	61	0.98	0.77	56	0.99	0.93	0.99	58.3
**4bpf**	1.27	1.01	6.53	1.32	4	6	0.44	20	0.69	126	0.96	0.75	25	0.99	0.73	0.84	52.7
**6fmb**	1.08	0.54	8.78	2.10	8	6	0.73	53	0.70	43	0.91	0.72	36	0.97	0.64	0.74	35.1
**5k2l**	0.77	0.29	9.28	3.32	10	5	0.66	25	0.63	18	0.89	0.73	34	0.96	0.83	0.87	38.0
**3k3v**	3.54	1.69	9.29	2.66	5	5	0.75	61	0.72	68	0.96	0.78	56	0.99	0.94	0.93	34.4
**3fdr**	1.13	1.06	7.85	2.49	7	6	0.70	84	0.75	111	0.94	0.75	65	0.98	0.96	0.96	168.2
**4qy7**	1.32	0.77	7.36	2.61	5	5	0.60	25	0.72	63	0.95	0.77	44	0.98	0.05	0.59	185.6
**3dkm**	3.57	3.61	12	3.27	4	5	0.68	19	0.78	49	0.97	0.80	203	0.99	0.86	0.86	65.4
	1.51	0.92	7.31	2.31	6	5	0.66	53	0.70	73	0.94	0.76	71	0.98	0.71	0.82	64.3

[a]RMSD deviation of the REMD simulation. [b]RMSD deviation of RCD ensemble. RMSD deviations consider using only the backbone atoms and are computed respect to the closest loop to the average structures sampled by REMD. [c]Number of eigenvectors needed to explain 90% of the variance obtained by REMD and RCD. [d]Corresponding similarity indexes and Z-scores obtained with 90% variance. In [e] and [f], equivalent scores obtained with the first 10 and 20 modes, respectively. [f]Pearson and Spearman correlations of the B-factors obtained by REMD and RCD approaches. [g]Required time in seconds for sampling the 10000 loops with RCD

Table S3. Comparative measures of the last (from 4-5 μs) and the previous last (from 3-4μs) nanosecond of the REMD simulation trajectories.

	[a]RMSD REMD 3-4μ		[b]RMSD REMD 4-5μ		90% variance				[e]10 first modes			[e]20 first modes			[f]B-Factor	
**PDB**	Avg	σ	Avg	σ	[c]3-5μ	[c]4-5μ	[d]$\gamma_{90\%}$	[d]$Z_{90\%}$	γ_{10}	Z_{10}	var	γ_{20}	Z_{20}	var	Pearson	Spearman
**2eaq**	1.60	1.35	1.52	1.29	3	2	0.93	422	0.86	59	0.97	0.95	407	0.99	0.98	0.99
**5w0g**	1.02	0.55	1.03	0.62	7	6	0.97	831	0.89	362	0.94	0.93	86	0.98	1.00	0.99
**2ns0**	0.69	0.2	0.66	0.22	9	9	0.94	247	0.94	247	0.91	1.00	584	0.97	1.00	0.98
**4dpb**	0.75	0.49	0.79	0.40	7	7	0.85	346	0.93	714	0.93	0.96	470	0.98	1.00	1.00
**5nod**	0.87	0.38	1.04	0.38	5	5	1.00	233	0.93	377	0.95	1.00	520	0.98	0.98	0.97
**6elm**	1.64	0.55	1.57	0.86	6	7	0.94	388	0.95	736	0.95	0.95	398	0.99	1.00	1.00
**3bv8**	0.50	0.15	0.52	0.16	14	13	0.99	354	0.99	807	0.84	0.96	596	0.94	0.99	0.99
**5e9p**	2.66	1.05	2.85	0.90	4	3	0.88	99	0.90	625	0.97	0.91	412	0.99	1.00	1.00
**4bpf**	1.49	1.06	1.27	1.01	4	4	0.80	75	0.88	141	0.96	0.96	91	0.99	0.99	0.98
**6fmb**	1.06	0.63	1.08	0.54	8	8	0.86	114	0.90	354	0.92	0.95	558	0.97	0.99	0.99
**5k2l**	0.72	0.4	0.77	0.29	10	10	0.90	234	0.91	246	0.89	0.97	193	0.96	0.99	1.00
**3k3v**	3.95	1.85	3.54	1.69	5	5	0.96	229	0.93	78	0.96	0.89	103	0.99	0.97	0.97
**3fdr**	1.26	1.43	1.13	1.06	5	7	0.83	188	0.96	280	0.96	0.92	191	0.98	0.98	0.99
**4qy7**	1.45	0.81	1.32	0.77	5	5	0.81	251	0.94	405	0.96	0.91	531	0.98	0.99	0.99
**3dkm**	5.53	2.69	3.57	3.61	6	4	0.79	133	0.84	92	0.95	0.86	112	0.99	0.99	0.99
	1.68	0.91	1.51	0.92	6	6	0.90	276	0.92	368	0.94	0.94	350	0.98	0.98	0.99

[a,b]Backbone RMSD deviation from the closest to the average loop structure sampled in the corresponding REMD simulation. [c]Number of eigenvectors needed to explain 90% of the variance. [d]Corresponding similarity indexes and Z-scores obtained with 90% variance. In [e] and [f], equivalent scores obtained with the first 10 and 20 modes, respectively. [f]Pearson and Spearman correlations of the B-factors obtained

Table S4. RMSDs of the initial REMD loop conformation and final morphed conformation obtained with the NMA constrained loop sampling respect to the target crystallographic structure.

	Indexes		Sequence	RMSD N-CA-C	
**PDB**	Start	End		Initial	Final
**2eaq**	12	36	NQTPGKSLDFGF	9.00	0.64
**5w0g**	24	37	LGGLTQAPGNPV	10.32	0.65
**2ns0**	2	9	VVDPRSARGPIR	8.33	0.50
**4dpb**	9	6	FDEDSIPSGVDA	11.63	0.38
**5nod**	1	26	VTTAPAGSVEGM	7.43	0.56
**6elm**	1	29	KLKGKPKDNGAI	5.13	0.64
**3bv8**	4	12	GNFEGITYPESFK	7.14	0.76
**5e9p**	3	33	TDQFSTDPNDWSRY	10.68	0.78
**4bpf**	1	15	ENPDIEIFEEGLLDA	10.45	0.96
**6fmb**	31	19	VEDSDANWQGGAVKY	12.83	0.55
**5k2l**	17	13	LNPGVNPARLQVGQV	15.64	0.63
**3k3v**	16	8	RLGSTPETLGINDIFI	15.60	0.51
**3fdr**	7	33	YENSVPEDLTVHVGDI	11.10	0.86
**4qy7**	11	46	GLSEIDNTDDLKRGEK	5.76	0.54
**3dkm**	59	32	RGLDWKWRDQDGSPQGEG	15.37	0.57
				10.42	0.64
